# Supplementary material for: Vector-borne diseases and the Syrian conflict: A systematic review of literature from Syria and neighbouring, refugee-hosting countries
Source: PLoS Negl Trop Dis. 2025 Nov 26;19(11):e0013721. doi: 10.1371/journal.pntd.0013721 (PMC12654875; doi:10.1371/journal.pntd.0013721)
Supplement: S1 Appendix — (DOCX) [file pntd.0013721.s001.docx]

S1 Appendix

***Table of search strategy terms across 4 chosen databases.***

| **Search Term** | **Medline** | **Embase** | **Global Health** | **Scopus** |
| --- | --- | --- | --- | --- |
| 1 | syria*.mp. or Syria/ | Syria.mp. or Syrian Arab Republic/ | Syria*.mp. or Syria/ | syria* |
| 2 | limit 1 to yr="2011 -Current" | limit 1 to yr="2011 -Current” | limit 1 to yr="2011 -Current" | ( syria* )  AND  PUBYEAR  >  2010 |
| 3 | (Turkey adj4 (refugee* or migrant*)).mp. [mp=title, abstract, original title, name of substance word, subject heading word, floating sub-heading word, keyword heading word, organism supplementary concept word, protocol supplementary concept word, rare disease supplementary concept word, unique identifier, synonyms] | (Turkey adj4 (refugee* or migrant*)).mp. [mp=title, abstract, heading word, drug trade name, original title, device manufacturer, drug manufacturer, device trade name, keyword, floating subheading word, candidate term word] | (Turkey adj4 (refugee* or migrant*)).mp. [mp=abstract, title, original title, broad terms, heading words, identifiers, cabicodes] | turkey  W/4  ( refugee*  OR  migrant* ) |
| 4 | (Lebanon adj4 (refugee* or migrant*)).mp. [mp=title, abstract, original title, name of substance word, subject heading word, floating sub-heading word, keyword heading word, organism supplementary concept word, protocol supplementary concept word, rare disease supplementary concept word, unique identifier, synonyms] | (Lebanon adj4 (refugee* or migrant*)).mp. [mp=title, abstract, heading word, drug trade name, original title, device manufacturer, drug manufacturer, device trade name, keyword, floating subheading word, candidate term word] | (Lebanon adj4 (refugee* or migrant*)).mp. [mp=abstract, title, original title, broad terms, heading words, identifiers, cabicodes] | lebanon  W/4  ( refugee*  OR  migrant* ) |
| 5 | (Jordan adj4 (refugee* or migrant*)).mp. [mp=title, abstract, original title, name of substance word, subject heading word, floating sub-heading word, keyword heading word, organism supplementary concept word, protocol supplementary concept word, rare disease supplementary concept word, unique identifier, synonyms] | (Jordan adj4 (refugee* or migrant*)).mp. [mp=title, abstract, heading word, drug trade name, original title, device manufacturer, drug manufacturer, device trade name, keyword, floating subheading word, candidate term word] | (Jordan adj4 (refugee* or migrant*)).mp. [mp=abstract, title, original title, broad terms, heading words, identifiers, cabicodes] | jordan  W/4  ( refugee*  OR  migrant* ) |
| 6 | (Iraq adj4 (refugee* or migrant*)).mp. [mp=title, abstract, original title, name of substance word, subject heading word, floating sub-heading word, keyword heading word, organism supplementary concept word, protocol supplementary concept word, rare disease supplementary concept word, unique identifier, synonyms] | (Iraq adj4 (refugee* or migrant*)).mp. [mp=title, abstract, heading word, drug trade name, original title, device manufacturer, drug manufacturer, device trade name, keyword, floating subheading word, candidate term word] | (Iraq adj4 (refugee* or migrant*)).mp. [mp=abstract, title, original title, broad terms, heading words, identifiers, cabicodes] | Iraq W/4  ( refugee*  OR  migrant* ) |
| 7 | 2 or 3 or 4 or 5 or 6 | 2 or 3 or 4 or 5 or 6 | 2 or 3 or 4 or 5 or 6 | 2 OR 3 OR 4 OR 5 OR 6 |
| 8 | Vector-borne disease*.mp. | vector borne disease*.mp. | Vector-borne disease*.mp. or vector-borne diseases.sh. | “vector-borne disease*” |
| 9 | Arbovirus.mp. or Arboviruses/ | arthropod vector/ or arthropod disease/ or arthropod.mp. | Arbovirus.mp. or exp arboviruses/ | arthropod-borne* |
| 10 | Arthropod-borne.mp. | Chikungunya virus/ or chikungunya/ or chikungunya.mp. | chikungunya virus disease/ or Chikungunya virus/ or Chikungunya.mp. | arbovirus |
| 11 | Chikungunya virus/ or Chikungunya Fever/ or Chikungunya.mp. | dengue hemorrhagic fever/ or Dengue.mp. or dengue/ or Dengue virus/ | Dengue.mp. or dengue/ or Dengue virus/ or dengue haemorrhagic fever/ | chikungunya |

| 12 | Dengue.mp. or Dengue/ or Dengue Virus/ | Lymphatic filariasis.mp. or exp lymphatic filariasis/ | Lymphatic filariasis.mp. or lymphatic filariasis/ | dengue |
| --- | --- | --- | --- | --- |
| 13 | Lymphatic filariasis.mp. or Elephantiasis, Filarial/ | 13  Rift valley fever.mp. or Rift Valley fever/ | Rift valley fever.mp. or Rift Valley fever/ | "breakbone fever*" |
| 14 | Rift valley fever.mp. or Rift Valley Fever/ | Yellow fever.mp. or yellow fever/ | Yellow fever.mp. or yellow fever/ | "Lymphatic filariasis" |
| 15 | Yellow Fever/ or Yellow fever.mp. | Zika virus/ or Zika fever/ or Zika.mp. | Zika virus/ or Zika fever/ or Zika.mp. | elephantiasis |
| 16 | Zika Virus Infection/ or Zika Virus/ or Zika.mp. | exp malaria falciparum/ or Malaria*.mp. | exp malaria/ or Malaria*.mp. | "rift valley fever*" |
| 17 | exp Malaria/ or Malaria*.mp. | Japanese encephalitis.mp. or Japanese encephalitis virus/ or Japanese encephalitis/ | West nile fever.mp. or West Nile fever/ | "yellow fever" |
| 18 | West Nile fever.mp. or West Nile Fever/ | West Nile fever.mp. or West Nile fever/ | Schistosomiasis.mp. or schistosomiasis/ | Zika |
| 19 | Schistosomiasis.mp. or Schistosomiasis/ | exp schistosomiasis/ or Schistosomiasis.mp. | Onchoceriasis.mp. or onchocerciasis.sh. | malaria* |
| 20 | Bilharziasis.mp. | exp onchocerciasis/ or Onchoceriasis.mp. | plague/ or Plague.mp. | Plasmodium |
| 21 | Onchocerciasis/ or Onchoceriasis.mp. or Onchocerciasis, Ocular/ | plague/ or Plague.mp. | Tungiasis.mp. or Tunga penetrans.od. | "west nile fever*" |
| 22 | Plague/ or Plague.mp. | Tungiasis.mp. or tungiasis/ | Typhus.mp. or louse-borne typhus/ or typhus fevers/ | schistosomiasis |
| 23 | Tungiasis.mp. or Tungiasis/ | typhus/ or Typhus.mp. | louse-borne relapsing fever/ or Louse-borne relapsing fever.mp. | bilharzia* |
| 24 | Typhus.mp. or Typhus, Epidemic Louse-Borne/ | Borrelia infection/ or Louse-borne relapsing fever.mp. | exp Leishmania/ or Leishmania*.mp. | "snail fever*" |
| 25 | Borrelia/ or Relapsing Fever/ or Louse-borne relapsing fever.mp. | Leishmania*.mp. or exp Leishmania/ | Sandfly fever.mp. or sandfly fever/ | onchoceriasis |
| 26 | Leishmania/ or Leishmania*.mp. | Phlebovirus/ or Sandfly fever.mp. or sandfly fever/ | Crimean-congo haemorrhagic fever.mp. or Crimean-Congo haemorrhagic fever/ | "river blindness" |
| 27 | Sandfly fever.mp. or Phlebotomus Fever/ | Crimean-congo haemorrhagic fever.mp. or Crimean Congo hemorrhagic fever/ | Lyme disease*.mp. or Lyme disease.sh. | “robles disease” |
| 28 | Crimean-congo haemorrhagic fever.mp. or Hemorrhagic Fever, Crimean/ | Lyme disease.mp. or Lyme disease/ | Relapsing fever.mp. or relapsing fever/ | plague |
| 29 | Lyme disease.mp. or Lyme Disease/ | Relapsing fever.mp. or Borrelia infection/ | Borreliosis.mp. or Borrelia.od. | "Yersinia pestis" |

| 30 | Relapsing fever.mp. or Borrelia burgdorferi Group/ or Relapsing Fever/ | rickettsiosis/ or Rickettsial disease*.mp. | Rickettsial disease*.mp. or rickettsial diseases.sh. or Rickettsia.od. | Tungiasis |
| --- | --- | --- | --- | --- |
| 31 | Borrelia/ or Borrelia Infections/ or Borreliosis.mp. or Tick-Borne Diseases/ | Rocky Mountain spotted fever/ or Spotted fever.mp. | tick-borne encephalitis/ or Tick-borne encephalitis.mp. or Tick-borne encephalitis virus.od. | "tunga penetrans" |
| 32 | Rickettsia Infections/ or Rickettsial disease*.mp. or Rickettsia/ | Q fever.mp. or Q fever/ | Tularaemia.mp. or tularaemia/ | chigoe  OR  nigua  OR  jigger  OR  "sand flea" |
| 33 | Spotted fever.mp. or Spotted Fever Group Rickettsiosis/ | Tick-borne encephalitis.mp. or tick borne encephalitis/ | Chagas disease.mp. or Chagas' disease.sh. | Typhus |
| 34 | Q fever.mp. or Q Fever/ | Tularaemia.mp. or tularemia/ | Sleeping sickness.mp. or African trypanosomiasis/ | "Rickettsia typhi" |
| 35 | Tick-borne encephalitis.mp. or Encephalitis, Tick-Borne/ | Chagas disease.mp. or Chagas disease/ | 8 or 9 or 10 or 11 or 12 or 13 or 14 or 15 or 16 or 17 or 18 or 19 or 20 or 21 or 22 or 23 or 24 or 25 or 26 or 27 or 28 or 29 or 30 or 31 or 32 or 33 or 34 | rickettsia* |
| 36 | Tularemia/ or Tularaemia.mp. | Sleeping sickness.mp. or African trypanosomiasis/ | 7 and 35 | "louse-borne relapsing fever*"  OR  "relapsing fever*" |
| 37 | Chagas disease.mp. or Chagas Disease/ | 8 or 9 or 10 or 11 or 12 or 13 or 14 or 15 or 16 or 17 or 18 or 19 or 20 or 21 or 22 or 23 or 24 or 25 or 26 or 27 or 28 or 29 or 30 or 31 or 32 or 33 or 34 or 35 or 36 |  | borrelia* |
| 38 | American trypanosomiasis.mp. | 7 and 37 |  | Leishmani* |
| 39 | Trypanosomiasis, African/ or Sleeping sickness.mp. |  |  | sandfl* |
| 40 | 8 or 9 or 10 or 11 or 12 or 13 or 14 or 15 or 16 or 17 or 18 or 19 or 20 or 21 or 22 or 23 or 24 or 25 or 26 or 27 or 28 or 29 or 30 or 31 or 32 or 33 or 34 or 35 or 36 or 37 or 38 or 39 |  |  | "sandfly fever*"  OR  "three day fever*"  OR  "phlebotomus fever*"  OR  pappataci |
| 41 | 8 and 40 |  |  | "crimean-congo haemorrhagic fever*"  OR  "crimean haemorrhagic fever*" |

| 42 |  |  |  | "lyme disease*" |
| --- | --- | --- | --- | --- |
| 43 |  |  |  | "relapsing fever*” |
| 44 |  |  |  | "spotted fever*"  OR  "Q fever*" |
| 45 |  |  |  | "tick borne encephalitis” |
| 46 |  |  |  | tularaemia  OR  "rabbit fever*"  OR  "Francisella tularensis" |
| 47 |  |  |  | "chagas disease"  OR  "American trypanosomiasis"  OR  "Trypanosoma cruzi" |
| 48 |  |  |  | "sleeping sickness"  OR  "african trypanosomiasis"  OR  "Trypanosoma brucei" |
| 49 |  |  |  | 8 or 9 or 10 or 11 or 12 or 13 or 14 or 15 or 16 or 17 or 18 or 19 or 20 or 21 or 22 or 23 or 24 or 25 or 26 or 27 or 28 or 29 or 30 or 31 or 32 or 33 or 34 or 35 or 36 or 37 or 38 or 39 or 40 or 41 or 42 or 43 or 44 or 45 or 46 or 47 or 48 |
| 50 |  |  |  | 7 and 49 |
