# Supplementary material for: Vector-borne diseases and the Syrian conflict: A systematic review of literature from Syria and neighbouring, refugee-hosting countries
Source: PLoS Negl Trop Dis. 2025 Nov 26;19(11):e0013721. doi: 10.1371/journal.pntd.0013721 (PMC12654875; doi:10.1371/journal.pntd.0013721)
Supplement: S2 Appendix — (DOCX) [file pntd.0013721.s002.docx]

S2 Appendix: Quality Assessment of Included Papers

| **First author, Year, Reference** | **Global Rating** | **Selection Bias** | **Study Design** | **Confounders** | **Data Collection Methods** | **Analyses** | |
| --- | --- | --- | --- | --- | --- | --- | --- |
| Kocarslan, 2013, 34 | 1 | 3 | N | 3 | 2 | 1 | 1 |
| Saroufim, 2014, 35 | 2 | 1 | N | 3 | 1 | 1 | 1 |
| Alawieh, 2014, 15 | 1 | 2 | N | 2 | 1 | 1 | 1 |
| Koltas, 2014, 36 | 2 | 2 | N | 3 | 1 | 1 | 1 |
| Turan, 2015, 37 | 1 | 2 | N | 1 | 1 | 1 | 1 |
| Inci, 2015, 38 | 2 | 2 | N | 3 | 1 | N/A | N/A |
| Zgheib, 2016, 39 | 2 | 2 | N | 3 | 1 | 1 | 1 |
| Dunya, 2016, 40 | 2 | 2 | N | 3 | 1 | 1 | 1 |

| **First author, Year, Reference** | **Global Rating** | | **Selection Bias** | **Study Design** | **Confounders** | | **Data Collection Methods** | | **Analyses** | |
| --- | --- | --- | --- | --- | --- | --- | --- | --- | --- | --- |
| Alsaied, 2017, 41 | 2 | 2 | | N | | 3 | 1 | 1 | | 1 |
| Ozkeklikci, 2017, 42 | 2 | 2 | | N | | 3 | 1 | 1 | | 1 |
| Eksi, 2017, 43 | 2 | 2 | | N | | 2 | 1 | 1 | | 3 |
| Hawat, 2017, 44 | 1 | 2 | | N | | 2 | 1 | 1 | | 1 |
| Kaman, 2017, 22 | 3 | 2 | | N | | 3 | 1 | 1 | | 3 |
| Beyhan, 2017, 45 | 3 | 3 | | N | | 2 | 1 | 1 | | 3 |
| Rehman, 2018, 27 | 1 | 2 | | N | | 2 | 1 | 1 | | 3 |
| Gurses, 2018, 46 | 2 | 2 | | N | | 2 | 1 | 1 | | 3 |
| Hajj, 2018, 47 | 3 | 2 | | N | | 3 | 1 | 1 | | 3 |
| Muhjazi, 2019, 28 | 1 | 2 | | N | | 3 | 1 | 1 | | 1 |

| **First author, Year, Reference** | **Global Rating** | | **Selection Bias** | | **Study Design** | | **Confounders** | | **Data Collection Methods** | | **Analyses** | | |
| --- | --- | --- | --- | --- | --- | --- | --- | --- | --- | --- | --- | --- | --- |
| Youssef, 2019, 48 | | 2 | | 2 | | N | | 2 | | 1 | | 1 | 1 |
| Amr, 2019, 49 | | 2 | | 2 | | N | | 3 | | 1 | | 1 | 1 |
| Eroglu, 2019, 50 | | 2 | | 2 | | N | | 3 | | 1 | | 1 | 1 |
| Özbilgin, 2019, 51 | | 2 | | 2 | | N | | 2 | | 1 | | 1 | 3 |
| Karaosmanoglu, 2019, 52 | | 2 | | 2 | | N | | 2 | | 1 | | 1 | 3 |
| Hijawi, 2019, 53 | | 2 | | 2 | | N | | 2 | | 1 | | 1 | 3 |
| Safadi, 2019, 54 | | 2 | | 1 | | Y | | 2 | | 1 | | 1 | 3 |
| Özbilgin, 2019, 55 | | 2 | | 2 | | N | | 3 | | 1 | | 1 | 1 |
| Karakus, 2019, 56 | | 2 | | 2 | | N | | 2 | | 1 | | 1 | 3 |
| Alhawarat, 2020 | | 2 | | 1 | | N | | 2 | | 2 | | 1 | 3 |
| Yentur Doni, 2020 | | 2 | | 1 | | N | | 2 | | 1 | | 1 | 3 |
| Bizri, 2021 | | 2 | | 1 | | N | | 2 | | 2 | | 1 | 3 |
| Hammoud, 2022 | | 2 | | 1 | | N | | 2 | | 2 | | 1 | 3 |
| Farah, 2023 | | 2 | | 1 | | N | | 2 | | 2 | | 1 | 3 |
| Özbilgin 2023 | | 2 | | 2 | | N | | 2 | | 1 | | 1 | 3 |
