# Supplementary material for: Vector-borne diseases and the Syrian conflict: A systematic review of literature from Syria and neighbouring, refugee-hosting countries
Source: PLoS Negl Trop Dis. 2025 Nov 26;19(11):e0013721. doi: 10.1371/journal.pntd.0013721 (PMC12654875; doi:10.1371/journal.pntd.0013721)
Supplement: Attachment — Submitted filename: Reviewer Recommendation.docx [file pntd.0013721.s005.docx]

Reviewer Recommendation and Comments for Manuscript Number PNTD-D-25-00006

The article is not sufficiently innovative, and similar work has been done in published articles in this field that provide more comprehensive results. On the other hand, the authors have mentioned the general term "vector-borne disease" in the title and text of the paper, meanwhile, it seems that the only disease present was leishmaniasis.

Tarnas M, Lassmann B, Desai A, Abbara A. **Impact of the Syrian conflict on vector-borne disease trends in Syria and neighboring countries: A one health ProMED analysis between 2003 to 2018**. International Journal of Infectious Diseases. 2020 Dec 1;101:210.

Karami M, Gorgani-Firouzjaee T, Chehrazi M. **Prevalence of cutaneous Leishmaniasis in the Middle East: a systematic review and meta-analysis.** Pathogens and Global Health. 2023 May 19;117(4):356-65.
