# Supplementary material for: Vector-borne diseases and the Syrian conflict: A systematic review of literature from Syria and neighbouring, refugee-hosting countries
Source: PLoS Negl Trop Dis. 2025 Nov 26;19(11):e0013721. doi: 10.1371/journal.pntd.0013721 (PMC12654875; doi:10.1371/journal.pntd.0013721)
Supplement: Attachment — Submitted filename: Response to Reviewers Comments VBD Review.docx [file pntd.0013721.s007.docx]

Dear Editors,

Many thanks for the reviewers’ comments. We have responded to these point and point and enclose a tracked and untracked version of the manuscript. We have also been through to strengthen the messages from this review, emphasizing that it makes a distinct contribution to the literature on vector borne diseases. We hope that you will consider these changes favorably.

Thank you

Aula Abbara

**Editor:**

Please, consider all reviewers' comments and modify the title so that it better reflects the content of this review.

Response:

We have considered all reviewers’ comments and we have changed the title which now reads ‘Vector-borne diseases and the Syrian Conflict: A Systematic Review of literature from Syria and neighbouring, refugee-hosting countries.’

**Journal Requirements:**

1) Please ensure that the CRediT author contributions listed for every co-author are completed accurately and in full.
   At this stage, the following Authors/Authors require contributions: John William Carew, Remi Simpson, Timesh D Pillay, Angel Desai, Richard Toalster, Pylin Parkes, and Aula Abbara. Please ensure that the full contributions of each author are acknowledged in the "Add/Edit/Remove Authors" section of our submission form.
   The list of CRediT author contributions may be found here: <https://journals.plos.org/plosntds/s/authorship#loc-author-contributions>

Response:

We have now completed the CRediT author contributions as follows:

JWC contributed to data curation, formal analysis, investigation, validation, writing – original draft preparation, and writing – review & editing.

RS contributed to data curation, formal analysis, methodology, visualisation, writing - original draft preparation.

TDP contributed to data curation, methodology, writing - original draft preparation.

AD contributed to data curation, methodology, writing - original draft preparation.

RT contributed to data curation, methodology, writing - original draft preparation; writing - review and editing

PP contributed to data curation, methodology, writing - original draft preparation.

AA contributed to conceptualization, methodology, project administration, supervision, and writing – review & editing.

**Reviewer:**

2) We ask that a manuscript source file is provided at Revision. Please upload your manuscript file as a .doc, .docx, .rtf or .tex. If you are providing a .tex file, please upload it under the item type u2018LaTeX Source Fileu2019 and leave your .pdf version as the item type u2018Manuscriptu2019.

**Response:**

We thank the reviewers for their comment and have provided a manuscript source file at revision.

**Reviewer:**

3) Please provide an Author Summary. This should appear in your manuscript between the Abstract (if applicable) and the Introduction, and should be 150-200 words long. The aim should be to make your findings accessible to a wide audience that includes both scientists and non-scientists. Sample summaries can be found on our website under Submission Guidelines:
<https://journals.plos.org/plosntds/s/submission-guidelines#loc-parts-of-a-submission>

**Response:**

We thank the reviewers for their comment and have included the following as an author summary:

Author Summary: The conflict in Syria has severely impacted public health infrastructure, leading to a rise in communicable diseases, particularly vector-borne diseases (VBDs) such as leishmaniasis. This systematic review examined 33 studies published between March 2011 and May 2024, focusing on Syria and neighboring refugee-hosting countries—Turkey, Lebanon, Jordan, and Iraq. Leishmaniasis emerged as the most commonly reported VBD among Syrians, with only one study addressing malaria. Reported cases of leishmaniasis increased over time and spread geographically, especially in refugee-hosting areas. However, barriers to healthcare access and poor disease surveillance suggest the true burden is likely underestimated. Most studies relied on health ministry data, while smaller studies highlighted critical issues such as healthcare inaccessibility. There is a notable gap in research on effective intervention strategies and the vectors responsible for disease transmission. Further investigation into vector control and broader VBD prevalence is essential to inform better outbreak response and public health planning in the region.

**Reviewer:**

4) Please upload all main figures as separate Figure files in .tif or .eps format. For more information about how to convert and format your figure files please see our guidelines: 
<https://journals.plos.org/plosntds/s/figures>

**Response:**

We thank the reviewers for their comment and have provided all main figures as separate Figure files in .tiff format.

**Reviewer:**

5) We notice that your supplementary Tables, and information are included in the manuscript file. Please remove them and upload them with the file type 'Supporting Information'. Please ensure that each Supporting Information file has a legend listed in the manuscript after the references list.

**Response:**

We thank the reviewers for their comment and have included all supplementary tables with legends in a Supporting Information file.

**Reviewer:**

6) Some material included in your submission may be copyrighted. According to PLOSu2019s copyright policy, authors who use figures or other material (e.g., graphics, clipart, maps) from another author or copyright holder must demonstrate or obtain permission to publish this material under the Creative Commons Attribution 4.0 International (CC BY 4.0) License used by PLOS journals. Please closely review the details of PLOSu2019s copyright requirements here: PLOS Licenses and Copyright. If you need to request permissions from a copyright holder, you may use PLOS's Copyright Content Permission form. Please respond directly to this email and provide any known details concerning your material's license terms and permissions required for reuse, even if you have not yet obtained copyright permissions or are unsure of your material's copyright compatibility. Once you have responded and addressed all other outstanding technical requirements, you may resubmit your manuscript within Editorial Manager. 

Potential Copyright Issues:
i) Figures 1, and 3. Please (a) provide a direct link to the base layer of the map (i.e., the country or region border shape) and ensure this is also included in the figure legend; and (b) provide a link to the terms of use / license information for the base layer image or shapefile. We cannot publish proprietary or copyrighted maps (e.g. Google Maps, Mapquest) and the terms of use for your map base layer must be compatible with our CC BY 4.0 license.Note: if you created the map in a software program like R or ArcGIS, please locate and indicate the source of the basemap shapefile onto which data has been plotted.
If your map was obtained from a copyrighted source please amend the figure so that the base map used is from an openly available source. Alternatively, please provide explicit written permission from the copyright holder granting you the right to publish the material under our CC BY 4.0 license.
If you are unsure whether you can use a map or not, please do reach out and we will be able to help you. The following websites are good examples of where you can source open access or public domain maps:
* U.S. Geological Survey (USGS) - All maps are in the public domain. ([http://www.usgs.gov](http://www.usgs.gov/))
* PlaniGlobe - All maps are published under a Creative Commons license so please cite u201cPlaniGlobe, [http://www.planiglobe.com](http://www.planiglobe.com/), CC BY 2.0u201d in the image credit after the caption. (<http://www.planiglobe.com/?lang=enl>)
* Natural Earth - All maps are public domain. (<http://www.naturalearthdata.com/about/terms-of-use/>).

**Response:**

We thank the reviewers for their comments and suggestions. The administrative boundaries and surface map of Figure 1 come from Open Data Soft (https://public.opendatasoft.com/explore/dataset/world-administrative-boundaries/map/?flg=en-us&location=5,34.57895,28.74023&basemap=jawg.light), and the license allows for academic use (<https://www.nationalarchives.gov.uk/doc/open-government-licence/version/3/>). The administrative boundaries of Figure 3 were obtained from GADM (https://gadm.org/data.html), and the license indicates that the data are freely available for academic use (<https://gadm.org/license.html>).

**Reviewer:**

ii) Appendix 2 contains a logo or branding. We are not permitted to publish this under our CC-BY 4.0 license, even with permission. We ask that you please remove or replace it.

**Response:**

Appendix 2 has been removed. The figure formerly labeled Appendix 3 has been renamed as Appendix 2.

**Reviewer:**
7) We note that your Data Availability Statement is currently as follows: "All data is available in the manuscript". Please confirm at this time whether or not your submission contains all raw data required to replicate the results of your study. Authors must share the “minimal data set” for their submission. PLOS defines the minimal data set to consist of the data required to replicate all study findings reported in the article, as well as related metadata and methods (<https://journals.plos.org/plosone/s/data-availability#loc-minimal-data-set-definition>).
For example, authors should submit the following data: 
1) The values behind the means, standard deviations and other measures reported;
2) The values used to build graphs;
3) The points extracted from images for analysis..
Authors do not need to submit their entire data set if only a portion of the data was used in the reported study.
If your submission does not contain these data, please either upload them as Supporting Information files or deposit them to a stable, public repository and provide us with the relevant URLs, DOIs, or accession numbers. For a list of recommended repositories, please see <https://journals.plos.org/plosone/s/recommended-repositories>. 
If there are ethical or legal restrictions on sharing a de-identified data set, please explain them in detail (e.g., data contain potentially sensitive information, data are owned by a third-party organization, etc.) and who has imposed them (e.g., an ethics committee). Please also provide contact information for a data access committee, ethics committee, or other institutional body to which data requests may be sent. If data are owned by a third party, please indicate how others may request data access.

**Response:**

We thank the reviewers for their comment. All data utilized is available in the manuscript. As this study was a narrative review, there are no quantitative measures reported.

**Reviewer:**

8) Please provide a completed 'Competing Interests' statement, including any COIs declared by your co-authors. If you have no competing interests to declare, please state "The authors have declared that no competing interests exist". Otherwise please declare all competing interests beginning with the statement "I have read the journal's policy and the authors of this manuscript have the following competing interests:"

**Response:**

The authors have declared that no competing interests exist.

**Reviewer:**
9) Please upload the PRISMA flowchart as Figure 1.

**Response:**

We thank the reviewer for their comment. The PRISMA flowchart has been uploaded as Figure 1.

**Reviewer:**

10) As required by our policy on Data Availability, please ensure your manuscript or supplementary information includes the following:
- A numbered table of all studies identified in the literature search, including those that were excluded from the analyses.
- For every excluded study, the table should list the reason(s) for exclusion.
- If any of the included studies are unpublished, include a link (URL) to the primary source or detailed information about how the content can be accessed.
- A table of all data extracted from the primary research sources for the systematic review and/or meta-analysis. The table must include the following information for each study:
  - Name of data extractors and date of data extraction
  - Confirmation that the study was eligible to be included in the review.
  - All data extracted from each study for the reported systematic review and/or meta-analysis that would be needed to replicate your analyses.
  - If data or supporting information were obtained from another source (e.g. correspondence with the author of the original research article), please provide the source of data and dates on which the data/information were obtained by your research group.
  - If applicable for your analysis, a table showing the completed risk of bias and quality/certainty assessments for each study or outcome.  Please ensure this is provided for each domain or parameter assessed. For example, if you used the Cochrane risk-of-bias tool for randomized trials, provide answers to each of the signalling questions for each study. If you used GRADE to assess certainty of evidence, provide judgements about each of the quality of evidence factor. This should be provided for each outcome.
  - An explanation of how missing data were handled.
This information can be included in the main text, supplementary information, or relevant data repository. Please note that providing these underlying data is a requirement for publication in this journal, and if these data are not provided your manuscript might be rejected.

**Response:**

We thank the reviewers for their comments. Table 3 has been amended to include the names of the names of data extractors and date of data extraction.

**Comments to the Authors:**

Please note that one of the reviews is uploaded as an attachment.

**Response:**

Thank you for your comments. We have copied the review that was included as an attachment into this document below.

**Reviewer:**

Reviewer #2: The objectives are clearly stated in the abstract and introduction, focusing on exploring the epidemiological burden and geographical distribution of VBDs in Syria and neighboring countries following the conflict. However, the study is a systematic review, and thus does not have a hypothesis in the same way as an original research study. This should be acknowledged.

Response: We have included the following “. Given that this is a systematic review, we have not made a hypothesis as our aim is to capture all relevant literature and undertake a narrative review.”

Reviewer:

The systematic review design is appropriate for addressing the objectives. PRISMA guidelines were followed, which enhances the study's rigor.

The population (Syrian refugees and host countries) is clearly described and relevant to the hypothesis.

As this is a systematic review, "sample size" refers to the number of studies included. The authors included 33 studies, which appears to be a reasonable number for a systematic review. The review's strength lies in synthesizing the available data, not in statistical power calculations.

Systematic reviews primarily involve narrative synthesis of data. The authors extracted data and summarized it narratively, which is appropriate. They also assessed the quality of included studies using the EPHPP tool, which is a recognized method.

Ethical approval is not applicable for a systematic review of published studies.

**Response:**

Thank you

**Reviewer:**

I believe the closer co-operation with local experts and refugee camps will bring more needed results, as are presented in the current study. Inclusion criteria should be more broad, authors are missing important VBD such as e.g. Crimean-Congo hemorrhagic fever. We similar and more detailed work was published in 2021 <https://www.ijidonline.com/article/S1201-9712(20)32169-X/pdf>

**Response:**

Thank you. The lead authors for this paper always work to support local experts/ researchers to undertake relevant projects on Syria. For this review, it started as a piece to inform future work with local experts. We have deliberately kept the inclusion criteria broad to capture as much about VBDs in Syria (and the main Syrian refugee-hosting countries.) A key part of this work is to highlight gaps - both for leishmaniasis for which there is the most literature and for other VBDs for which the literature is almost non-existent. Two of the authors have contributed to the IJID article with ProMed which has been recommended and we have now included this paper in the references. For CCHF, there is no academic publication on this in Syria or Syrian refugees as, though it is present in Iraq and Turkey in particular, given limited diagnostics in Syria, it is not mentioned. We have inserted the following to note this gap:

“We note for example, an absence of literature in Syria or among Syrian refugees in neighbouring countries on Crimean Congo Haemorrhagic Fever (CCHF,) and other tick-borne infections e.g. rickettsial infections or arboviruses. For CCHF, this is important given its prevalence in Turkey and Iraq, both of which share borders with Syria. This review forms a baseline to understand the current availability of data on VBDs (or lack thereof) and, given the impacts of climate change how VBDs may emerge or re-emerge; this would also include the impact on leishmaniasis, whose distribution in Syria has changed over the course of the conflict and is itself, impacted by climate change.”

**Reviewer:**

Results

Reviewer #2: Yes, the results section clearly presents the findings of the literature review, including study characteristics, VBD-related factors, causes for increased burden, and barriers to healthcare access, consistent with the stated methods.

Results are clearly presented, but Tables 2 and 3 could be simplified for better readability.

Figures 1–3 and tables are informative but would benefit from higher resolution and clearer labels.

Reviewer #3: Analysis match the plan and results are clearly presented.

**Response:**

We thank the reviewer for their important comments and have amended Figures 1-3 based on the comments of other reviewers. We believe that the revised figures uploaded in .tiff form have higher resolution.

**Reviewer:**

Conclusions

Reviewer #2: Conclusions are supported by the data, emphasizing leishmaniasis as the primary VBD and highlighting gaps in research.

The exclusion of grey literature and non-English studies is noted, but the authors should discuss how this might bias findings.

Yes, the public health relevance is evident throughout the manuscript. The review highlights the importance of understanding and addressing VBDs in conflict settings, with clear implications for healthcare planning and response.

Reviewer #3: Conclusions are supported by the presented data and public health relevance is addressed. However, the review is only partial, and the major limitation is the exclusion of non-English language journals and the lack of cooperation with local experts.

**Response:**

We have discussed the possibility that not including non-academic or non-English language papers may influence the results of the review. However, the lead authors are Arabic speakers and have searched in Arabic to see if relevant academic papers are identified; they have found none. This is likely due to the fact that most research on Syria is published in English-language journals, especially during the conflict. We do not have Turkish speakers as part of the authorship but in Turkey, academic outputs are also usually in English (or bilingual.) We have inserted a comment about this in the Strengths and Limitations section.

**Summary and General Comments**

Use this section to provide overall comments, discuss strengths/weaknesses of the study, novelty, significance, general execution and scholarship. You may also include additional comments for the author, including concerns about dual publication, research ethics, or publication ethics. If requesting major revision, please articulate the new experiments that are needed.

Reviewer:

Reviewer #3: The paper title “Vector-borne diseases and the Syrian Conflict: A Systematic Review” was focused on Syria and neighbouring, refugee-hosting countries, but we know a lot of refugees are hosted by EU member states such as Germany. To be accurate, I suggest the change of the title to, “Vector-borne diseases and the Syrian Conflict: A Systematic Review focused on Syria and neighbouring, refugee-hosting countries”.

Response:

We have changed the title to: “Vector-borne diseases and the Syrian Conflict: A Systematic Review of literature from Syria and neighbouring, refugee-hosting countries.” We agree that there are currently large numbers of Syrian refugees in Europe (particularly Germany,) the number of leishmaniasis cases reported are comparatively few compared to the refugee-hosting countries surrounding Syria.

Reviewer:

Thanks to the inclusion criteria, the authors analysed 33 studies describing mainly leishmaniasis. The manuscript is well written with minor formal mistakes. However, I disagree with the authors' statement about the limitations of their study. Authors excluded non-English language academic publications and according to them, “this is unlikely to have a significant influence on the key information identified as most key, academic literature related to this topic is published in English." A lot of important data has been published only in local journals written in the native language of practitioners from a non-academic environment. These are people working directly with refugees. However, as non-scientifically oriented health workers, they usually tend to publish their data only for the local experts in the local journals. Journals are usually published in the native language and are not listed in databases. We can see this trend even in EU countries. I believe this is the main limitation of the current study, describing not more than the already available data without direct communication with refugee camps. The main advantage of the current manuscript for me is not the need to read 33 studies, but only one, which is fine and fits the scope of the journal. However, I do not see any further scientific impact, and I will appreciate the authors admitting it, as a minimum, in the Strengths and limitations section. Moreover, similar work was published in 2021, <https://www.ijidonline.com/article/S1201-9712(20)32169-X/pdf>.

Response:

This is an important point; we based our statement on the paucity of academic publications in Arabic or in Syria-led Arabic language journals. We hand searched in Arabic to check this and found no reports of leishmaniasis. We did the same in Turkish. In general, for Syrian academic publications, these are done in English; an example of this is the Avicenna Journal of Medicine which is a Syrian led journal based in the United States which is published in English. We have however removed the phrase ‘this is unlikely to have a significant influence on the key information identified as most key, academic literature related to this topic is published in English.’ With regards to scientific impact, we have included the following ‘Given the nature of the publications, a strength of this literature is its summation of findings on this topic together with gaps in the literature.’

[NOTE: If reviewer comments were submitted as an attachment file, they will be attached to this email and accessible via the submission site. Please log into your account, locate the manuscript record, and check for the action link "View Attachments". If this link does not appear, there are no attachment files.]

**Figure resubmission:**

While revising your submission, please upload your figure files to the Preflight Analysis and Conversion Engine (PACE) digital diagnostic tool, <https://pacev2.apexcovantage.com/>. PACE helps ensure that figures meet PLOS requirements. To use PACE, you must first register as a user. Registration is free. Then, login and navigate to the UPLOAD tab, where you will find detailed instructions on how to use the tool. If you encounter any issues or have any questions when using PACE, please email PLOS at [figures@plos.org](mailto:figures@plos.org). Please note that Supporting Information files do not need this step. If there are other versions of figure files still present in your submission file inventory at resubmission, please replace them with the PACE-processed versions.

Response:

The figures have been reuploaded as PACE compatible .tif files.

**Reproducibility:**

To enhance the reproducibility of your results, we recommend that authors of applicable studies deposit laboratory protocols in [protocols.io](http://protocols.io/), where a protocol can be assigned its own identifier (DOI) such that it can be cited independently in the future. Additionally, PLOS ONE offers an option to publish peer-reviewed clinical study protocols. Read more information on sharing protocols at <https://plos.org/protocols?utm_medium=editorial-email&utm_source=authorletters&utm_campaign=protocols>

Reviewer:

Reviewer Recommendation and Comments for Manuscript Number PNTD-D-25-00006

The article is not sufficiently innovative, and similar work has been done in published articles in this field that provide more comprehensive results. On the other hand, the authors have mentioned the general term “vector-borne disease” in the title and text of the paper, meanwhile, it seems that the only disease present was leishmaniasis.Tarnas M, Lassmann B, Desai A, Abbara A. Impact of the Syrian conflict on vector-borne disease trends in Syria and neighboring countries: A one health ProMED analysis between 2003 to 2018. International

Journal of Infectious Diseases. 2020 Dec 1;101:210.

Karami M, Gorgani-Firouzjaee T, Chehrazi M. Prevalence of cutaneous Leishmaniasis in the Middle East: a systematic review and meta-analysis. Pathogens and Global Health. 2023 May 19;117(4):356-65.

Authors: The aim of this paper was to review all academic literature on vector-borne diseases in Syria and in the surrounding Syrian-refugee hosting countries. The paucity of data on vector-borne diseases academic literature related to humans is important, particularly as Syria and other countries in the region will likely face a change in the epidemiology of VBDs related to climate chante. Despite the near absence of data on other VBDs, we decided to keep to our initial intention. The paper by Tarnas et al takes a different approach; it relies on ProMed reports only - rather than broader academic literature and it differs from Karami’s which focuses mainly on CL prevalence whereas we aimed to capture broader literature which also relates to healthcare access and VBDs more generally. As such, the contribution of the paper is novel when compared to previously published literature. We see this as a useful baseline highlighting 1. The almost near absence of mention on non-leishmaniasis VBDs and 2. The poor academic exploration even for leishmaniasis. Additionally, we emphasise some of the aspects which are not covered in other literature, particularly around the causative vectors, barriers to healthcare access and living conditions of refugees which can impact VBDs.

This review therefore provides a useful summary of existing literature and the basis for future exploration, emphasising the need to better characterize the state of VBDs in Syria and the main surrounding refugee-hosting countries. We see it as a ‘call for action,’ highlighting the lack of rigorous exploration of emerging and re-emerging VBDs in an important context and one where attention should be paid given the risk of outbreaks. We have inserted the following paragraph to emphasise this argument:

“We note for example, an absence of literature in Syria or among Syrian refugees in neighbouring countries on Crimean Congo Haemorrhagic Fever (CCHF,) and other tick-borne infections e.g. rickettsial infections or arboviruses. For CCHF, this is important given its prevalence in Turkey and Iraq, both of which share borders with Syria. This review forms a baseline to understand the current availability of data on VBDs (or lack thereof) and, given the impacts of climate change how VBDs may emerge or re-emerge; this would also include the impact on leishmaniasis, whose distribution in Syria has changed over the course of the conflict and is itself, impacted by climate change.^77,78^”
